# Eravacycline, an antibacterial drug, repurposed for pancreatic cancer therapy: insights from a molecular-based deep learning model

**DOI:** 10.1093/bib/bbae108

**Published:** 2024-04-21

**Authors:** Adi Jabarin, Guy Shtar, Valeria Feinshtein, Eyal Mazuz, Bracha Shapira, Shimon Ben-Shabat, Lior Rokach

**Affiliations:** Department of Clinical Biochemistry and Pharmacology, Ben-Gurion University of the Negev (BGU), P.O.B. 653, Beer-Sheva 8410501, Israel; Department of Information Systems and Software Engineering, Ben-Gurion University of the Negev, P.O.B. 653, Beer-Sheva 8410501, Israel; Department of Clinical Biochemistry and Pharmacology, Ben-Gurion University of the Negev (BGU), P.O.B. 653, Beer-Sheva 8410501, Israel; Department of Information Systems and Software Engineering, Ben-Gurion University of the Negev, P.O.B. 653, Beer-Sheva 8410501, Israel; Department of Information Systems and Software Engineering, Ben-Gurion University of the Negev, P.O.B. 653, Beer-Sheva 8410501, Israel; Department of Clinical Biochemistry and Pharmacology, Ben-Gurion University of the Negev (BGU), P.O.B. 653, Beer-Sheva 8410501, Israel; Department of Information Systems and Software Engineering, Ben-Gurion University of the Negev, P.O.B. 653, Beer-Sheva 8410501, Israel

**Keywords:** pancreatic ductal adenocarcinoma, machine learning, drug repurposing, eravacycline

## Abstract

**Background:**

Pancreatic ductal adenocarcinoma (PDAC) remains a serious threat to health, with limited effective therapeutic options, especially due to advanced stage at diagnosis and its inherent resistance to chemotherapy, making it one of the leading causes of cancer-related deaths worldwide. The lack of clear treatment directions underscores the urgent need for innovative approaches to address and manage this deadly condition. In this research, we repurpose drugs with potential anti-cancer activity using machine learning (ML).

**Methods:**

We tackle the problem by using a neural network trained on drug–target interaction information enriched with drug–drug interaction information, which has not been used for anti-cancer drug repurposing before. We focus on eravacycline, an antibacterial drug, which was selected and evaluated to assess its anti-cancer effects.

**Results:**

Eravacycline significantly inhibited the proliferation and migration of BxPC-3 cells and induced apoptosis.

**Conclusion:**

Our study highlights the potential of drug repurposing for cancer treatment using ML. Eravacycline showed promising results in inhibiting cancer cell proliferation, migration and inducing apoptosis in PDAC. These findings demonstrate that our developed ML drug repurposing models can be applied to a wide range of new oncology therapeutics, to identify potential anti-cancer agents. This highlights the potential and presents a promising approach for identifying new therapeutic options.

## INTRODUCTION

With minimal treatment options, pancreatic ductal adenocarcinoma (PDAC) is a devastating disease. This cancer is recognized as one of the deadliest malignancies, and it is the leading cause of cancer-related deaths in Western countries [1, 2]. The presence of multiple changes in signaling pathways may partially explain some of this cancer’s resistance mechanisms [3]. Pancreatic cancer patients’ survival rate is estimated to average 5 years at most [4]. Chemotherapy, radiation and surgery are widely used but do not result in significant improvements in clinical outcomes [5]. The absence of effective treatment options underscores the urgency for new approaches to treat and manage this deadly disease.

Repurposing drugs that have already been approved by the Food and Drug Administration (FDA) for other indications has become a widely accepted approach for discovering new anti-cancer drugs, reducing costs and eliminating the need for toxicological tests. An existing drug may have a higher success rate than a potential drug in the FDA’s new chemical entity track [6]. A deep learning approach for identifying and predicting new indications for existing drugs was extended to other areas [7–9]. In the case of pancreatic cancer, where the mechanisms of the disease remain unclear, this approach may be extremely valuable. Drug reuse for PDAC has received increasing attention in recent years; however, research in this area has mainly been driven by hypotheses based on the overlap between an existing pharmacological mechanism of action and the causes of the disease [9, 10]. While some of the drugs proposed showed promising anti-cancer activity, few successes have been reported.

As drug databases have grown, machine learning (ML)-based approaches for changing a drug’s designation have emerged. These tools identify new drug–disease interactions. ML-based approaches can then be optimized to repurpose a drug [11]. Disadvantages of existing drug repurposing tools include the facts that they are usually not disease-specific and they sometimes include data on drug mechanisms and pathways obtained from diverse biological frameworks that are not available for the relevant drugs. Tools that predict specific properties or activity based on the chemical structure may result in more accurate predictions.

Based on the recent success in identifying halicin, a new antibiotic, using ML [9], in our study, we trained the same message passing neural network (NN) (Chemprop) to predict the anti-cancer activity of small molecules [12], using drug data collected from various sources (e.g. DrugBank, ClinicalTrials.gov). In addition to analyzing the chemical structure of the drugs, we extended this anti-cancer prediction model to consider drug–target interaction (DTI) and drug–drug interaction (DDI) information and demonstrated the resulting improvement in the model’s accuracy. We used this model to predict the anti-cancer activity of chemical structures and drugs that have not been tested in clinical trials for cancer; specifically, we used the model to predict the anti-cancer activity of all FDA-approved molecules as a means of identifying approved molecules with unknown anti-cancer potential.

Analyzing the predictions of the ML anti-cancer model for the set of FDA-approved drugs, three antibacterial drugs from the tetracycline family were found to have high anti-cancer activity scores: eravacycline, tigecycline and omadacycline (listed in order of their ranking). While eravacycline and omadacycline have never been tested for their potential anti-cancer activity, tigecycline, which was approved as an antibiotic in 2005 [13, 14], demonstrated possible anti-cancer activity and, in the case of PDAC, exerted its action via downregulating of CCNE2 [15]. Eravacycline and omadacycline were developed and approved in 2018 and showed excellent antibacterial activity [16, 17]. Drugs in the tetracycline family are broad-spectrum antimicrobial agents widely used in human medicine [13]. They are also used to treat a variety of diseases and disorders, including cancer and inflammation [14].

Recent research has suggested that the composition and diversity of bacterial communities in various body organs may play a role in the development of certain types of cancer. However, the exact mechanisms by which bacterial communities influence cancer development are still not fully understood [18]. For example, it has been found that changes in the gut microbiome may contribute to the development of colorectal cancer [19]. More research is needed to fully understand the complex interactions between bacterial communities and cancer development [20].

This study aims to train a Chemprop NN to predict the anti-cancer activity of FDA approved drugs, focusing on small molecules; the predictions are then validated in *in vitro* experiments. We investigated the anti-cancer activity of tetracyclines, according to the models’ predictions, and we identified eravacycline, a synthetic halogenated tetracycline-class antibiotic, as a leading candidate for repurposing for the treatment of PDAC.

## MATERIALS AND METHODS

**Figure 1:**
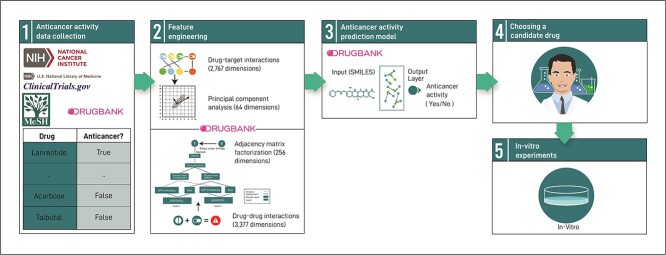
**Repurposing drugs with potential anti-cancer activity using machine learning**: (1) a labeled dataset is compiled from DrugBank, cancer.com, ClinicalTrials.gov and MeSH. (2) Dimensionality of drug-target and drug–drug interactions is reduced, and this information is utilized as tabular features. (3) A message-passing NN is trained to identify molecules with potential anti-cancer activity, using molecule structure and tabular features as input. (4) A pharmacologist selects promising candidates based on the model’s output. (5) The anti-cancer activity of these selected candidates is validated through *in vitro* experiments.

An overview of our methodology is presented in Figure 1. We collected anti-cancer training data from various sources (Figure 1.1). Then, we performed a feature engineering process (Figure 1.2) and trained an NN to rank drugs with anti-cancer potential (Figure 1.3). Finally, candidates were selected by a pharmacologist (Figure 1.4) and validated in *in vitro* experiments (Figure 1.5). The methodology’s phases are described in detail in the subsections that follow.

### Anti-cancer activity data collection

To train the anti-cancer activity prediction model, we collected a set of positive drugs consisting of 451 molecules with known anti-cancer activity from DrugBank 5.1.8, ClinicalTrials.gov and cancer.com, along with the drugs’ associated MeSH terms (Figure 1.1). About 918 negative drugs without anti-cancer activity were curated from the list of FDA-approved drugs after filtering out drugs that were already examined in cancer-related clinical trials (as reported in ClinicalTrials.gov) or drugs that are chemically like those drugs. In this research, we utilized the chemical similarity measure used on the DrugBank website (www.drugbank.ca); the default threshold of 0.7 was used. Afterward, a pharmacologist manually validated and corrected the list of positive and negative drugs collected by observing all the data sources and a textual description in DrugBank (see also Supporting Information, SI, Section S1). SI Figure S1 shows a t-distributed stochastic neighbor embedding (t-SNE) analysis of the representation of our entire dataset.

### Training anti-cancer activity prediction model

The set of drugs collected was used to train a Chemprop model to predict the molecules’ anti-cancer potential [12] (Figure 1.3). The model was trained to solve a binary classification problem. When predicting, the model assigns a continuous score quantifying the certainty of the prediction for each molecule. We used an ensemble of two Chemprop models.

Chemprop and the baselines presented below analyze the molecule structure via their simplified molecular input line entry system representation. In this research, we mainly analyze approved or near-approved drugs; these drugs can be represented by quality data. We exploit this fact by assessing the contribution of additional drug features after addressing their sparsity (Figure 1.2): (1) DTI information, which was obtained from DrugBank 5.1.8 and compressed, using principal component analysis (PCA), from a binary matrix of 2767 targets and 5857 drugs to a 64-dimensional representation of the drugs. The PCA processes decreased the number of dimensions from 2767 to 64 while retaining 45% of the variance. (2) DDI information (also obtained from DrugBank 5.1.8), originally represented by a rectangular binary matrix of 3377 drugs and reduced to a 256-dimensional compressed representation using adjacency matrix factorization with propagation (AMFP) [21], which performs factorization-like compression on the matrix. Missing information was filled in based on the chemically most similar single drug; similarity was calculated as described in the previous subsection. For molecules that have no similar molecules using DrugBank’s default threshold of 0.7, we used the average DTI and DDI information (see also SI, Sections S2–S4).

### Evaluation

We compare different set of features used with Chemprop. Additionally, we evaluate a few baseline models:

(i) ChemBERTa transformers-based molecular property prediction model, which was pretrained on PubChem by the authors of the original ChemBERTa paper [22] and fine-tuned for anti-cancer classification by the authors of the current paper.
(ii) eXtreme Gradient Boosting model trained on the Morgan fingerprints of the drugs.
(iii) Support vector machine (SVM) model trained on the Morgan fingerprints of the drugs.

The model was evaluated using a balanced scaffold-based split, which was repeated three times. The area under the receiver operating characteristic curve (AUC) served as the primary evaluation metric for the proposed models, but we also present the results based on the area under the precision–recall curve (AUPR), accuracy, Cohen’s kappa and the Matthews correlation coefficient (MCC) metrics.

### Identifying and selecting repurposable drug candidates

Our research represents a collaborative effort between pharmacologists and computational teams, aimed at leveraging the strengths of both disciplines in the drug discovery process. The integration of domain expertise and knowledge with advanced ML tools has proven to be instrumental in the identification and selection of potential drug candidates [23]. The anti-cancer model assessed DrugBank 5.1.8 drugs, assigning each an anti-cancer activity score, ranking them accordingly. In addition to the ranking score and commercial availability, the selection process is further refined through the active involvement of pharmacologists. This crucial step involves a meticulous evaluation of various factors to ensure the identification of not only high-ranking candidates but also those aligning with key pharmacological criteria. For instance, the inclusion of specific pharmacokinetic and pharmacodynamic parameters, examination of potential side effects and analysis of relevant biological pathways were informed by the expertise of our pharmacological team. This collaborative process ensured that the selected candidates not only met computational criteria but also demonstrated pharmacological efficacy and safety.

### Laboratory methods and experimental protocols

In this study, all laboratory methods, such as cell culturing, proliferation and migration assays, determination of half maximal inhibitory concentration (IC50), flow cytometry analyses, protein extraction and Western blot analysis, were conducted using established protocols. The detailed procedures can be found in SI, Sections S5–S12.

### Data analysis and statistical methodology

Data analysis utilized GraphPad Prism v5.01 (GraphPad Software, San Diego, CA, USA). Two-sample means were compared via Student’s *t*-test; multiple group comparisons used one-way analysis of variance (ANOVA) with Tukey’s *post hoc* test (^*^P < 0.05, ^*^^*^P < 0.01, ^*^^*^^*^P < 0.001). Experiments, in triplicate, determined mean ± standard deviation for statistical significance (*P* < 0.05).

## RESULTS

### *In silico* candidate identification

Table 1 presents the performance of the anti-cancer prediction baselines and models used in this research. We report an AUC of 0.82 for a model that only considers the drug’s molecule structure; however, the best-performing model was also trained using the drugs’ DTI and DDI information, and the enhanced model obtained an AUC of 0.92 (refer to SI, Figure S2). SI, Table S1 shows the confusion matrix for the best-performing model [24]. Eravacycline was ranked 17th out of the 1371 ranked molecules. We gave the drug higher priority due to the known anti-cancer activity of tetracyclines [25] and its commercial availability.

**Table 1:** **Average Results of Chemprop Model with Additional Drug Features, Including Baseline Models.** Average results of a Chemprop model trained on the molecular structure of the drugs for different combinations of additional drug features. The table also shows the results for the baseline models. RDKIT 2D features are calculated by Chemprop using functionality found in RDKIT software

Additional DRUG features	AUC	AUPR	Accuracy	Cohen’s kappa	MCC
Chemprop, DDI + DTI	0.92	0.90	0.88	0.72	0.72
Chemprop, DDI	0.90	0.85	0.86	0.68	0.69
Chemprop, DTI	0.86	0.78	0.80	0.55	0.55
Chemprop, RDKit	0.84	0.73	0.80	0.55	0.55
Chemprop, no features	0.82	0.73	0.76	0.47	0.47
Chemberta, fine-tuned	0.77	0.65	0.75	0.43	0.43
XGB, fingerprint	0.77	0.67	0.74	0.38	0.39
SVM, fingerprint	0.83	0.75	0.78	0.52	0.52

### Pharmacologist-driven drug candidate selection

The collaborative effort between computational and pharmacological teams in this selection process adds a layer of expertise that goes beyond the quantitative assessments provided by the AI model. In the process of selecting candidate drugs for further investigation, multiple factors were carefully considered to ensure a comprehensive and informed approach. Drugs that had already shown documented anti-cancer activity were excluded from the list to focus on novel potential candidates. For instance, trofosfamide (ranked 15th) and fenretinide (ranked 34th) showed documented anti-cancer activity [26, 27] joining the training set for model refinement, excluding them from *in vitro* testing. Eravacycline (ranked 17th among 1352 approved molecules) stood out due to untapped oncological exploration. Commercially available drugs took precedence for further scrutiny (Figure 1.5). Additional elements played a role in refining our drug selection process, encompassing various considerations such as the pharmacologist’s involvement in candidate selection. This involvement spans assessments of biological relevance, experimental feasibility and risk assessment (see also SI, Section S13).

### Effect of tetracycline derivatives on human breast, lung, colon and PDAC carcinoma cell viabilities

To investigate the effect of tetracycline derivatives on cell proliferation in human breast, lung, colon and PDAC cells, we treated all cell lines (MCF-7, A549, HT-29, BxPC-3 and AsPC-1) with different concentrations of tigecycline, omadacycline and eravacycline (1, 2, 5, 10, 25 and 50 μM), with medium used as a control, for 72 h. Then, The growth inhibition rate was determined using the tetrazolium dye, 2,3-bis-(2-methoxy-4-nitro-5-sulphenyl)-(2H)-tetrazolium-5-carboxanilide (XTT)

**Figure 2:**
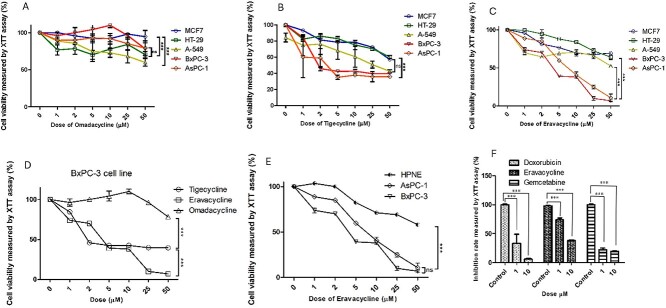
**Eravacycline suppresses cell growth and proliferation of pancreatic cancer cells**. (A), tigecycline (B), and eravacycline (C) in AsPC-1, BxPC-3, A-549, MCF-7, HT-29 cells for 72 h. (D) tigecycline, eravacycline, and omadacycline anti-cancer activity in the BxPC-3 cell line. (E) Inhibition rates of two human PDAC cell lines and one human normal pancreatic cell line (HPNE) treated with increasing concentrations of eravacycline for 72 h. (F) Inhibition rates of BxPC-3 cell line treated with increasing concentrations of eravacycline, doxorubicin, and gemcitabine for 72 h. Cell viability was determined by performing an XTT assay, which was performed to measure the IC50 values. At least three independent experiments were conducted. All data are shown as the mean ± SD. A comparison between multiple groups was performed using a one-way ANOVA. ^*^^*^^*^P < .001. ns, not significant. P-value <.05 was considered statistically significant.

The results show that treatment with increasing concentrations of omadacycline had no significant effect on cell survival (Figure 2A). The treatment of all cell lines with tigecycline for 72 h suppressed the proliferation of human PDAC (BxPC-3, AsPC-1) and A549 cell lines moderately in a concentration-dependent manner by up to 60–65%, while no significant effect was observed in the MCF-7 and HT-29 cell lines (Figure 2B). Treatment of the cells with eravacycline for 72 h suppressed the proliferation of human PDAC cells (BxPC-3, AsPC-1) in a concentration-dependent manner by up to 90–93% (Figure 2C). When administered at a concentration of 25 μM eravacycline was able to reduce the human BxPC-3 cells’ viability more effectively than 25 μM of tigecycline (a 93% versus 60% reduction, respectively, where *P* = 0.001). No significant effect was observed for eravacycline in the MCF-7, A549 and HT-29 cell lines (Figure 2C). These results indicate that both PDAC cell lines had a significant reduction in cell survival in a dose-dependent manner following treatment with eravacycline (Figure 2C). The IC50 of eravacycline on the BxPC-3 and AsPC-1 cells was 3.57 and 7.07 μM, respectively (Figure 2C). In contrast, tigecycline needed 9.9 and 4.58 μM to inhibit 50% of the BxPC-3 and AsPC-1 cells, respectively, at the same time point (Figure 2B).

After 72 h of treatment, eravacycline was shown to significantly decrease the BxPC-3 cells’ growth by 90% (*P* < 0.01) and 93% (*P* < 0.001) at 25 and 50 μM concentrations, respectively (Figure 2C), whereas it caused a 75% (*P* < 0.05) and 89% (*P* < 0.001) decrease in AsPC-1 cell growth at the same concentrations (Figure 2C). The BxPC-3 cell line, which was relatively more sensitive to eravacycline, was selected for further study. to compare the effect of eravacycline against pancreatic cancer cells to that against normal cells, we examined the effect of eravacycline on cell proliferation in one human normal pancreatic cell (HPNE cell line). The results revealed that HPNE cell was the most insensitive cell to eravacycline (Figure 2E). To compare eravacycline’s activity against BxPC-3 cancer cells to that of chemotherapeutic agents, we examined the anti-cancer activity of doxorubicin and gemcitabine. After 72 h of treatment, doxorubicin significantly decreased BxPC-3 cells’ growth by 67% (*P* < 0.001) and 94% (*P* < 0.001) at 1 and 10 μM concentrations, respectively (Figure 2F). However, gemcitabine significantly inhibited cell growth in the BxPC-3 cell line at all concentrations. The percentage of BxPC-3 cell growth inhibition was 77% and 80% at 1 and 10 μM, respectively (Figure 2F).

These results indicate that eravacycline significantly inhibited cell proliferation of BxPC-3 cells dose-dependently, and it was more cytotoxic to PDAC cells than tigecycline or omadacycline (Figure 2D). The results also show that the BxPC-3 cell line was the most sensitive to eravacycline, which was relatively less effective in breast (MCF-7), lung (A549) and colon (HT-29) cancer cell lines.

### Effect of eravacycline on the inhibition of cell migration in PDAC cells

**Figure 3:**
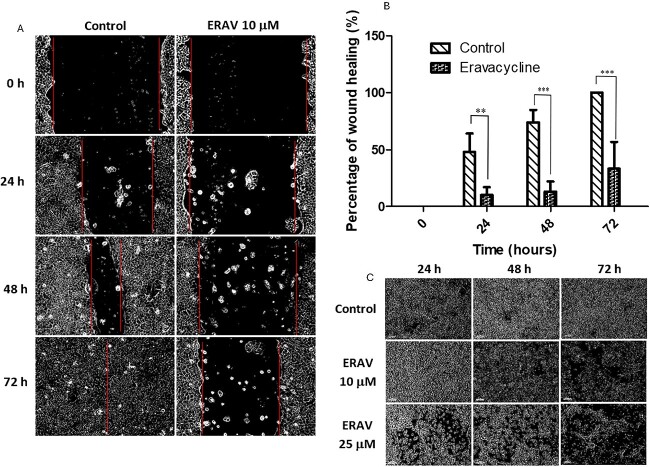
**Eravacycline inhibits cell migration in human PDAC cell lines**.(A) Migration assessment evaluated by wound healing assay of BxPC-3 cells after cultured in medium only (control) or medium with 10 μM of eravacycline for the indicated time. Scale bar, 100 μm. (B) The effect of 10 μM of eravacycline on wound closure in BxPC-3 cells. (c) Changes in colony appearance in BxPC-3 cells after cultured in medium only (control) or different concentrations of eravacycline for 24-72 h. At least three independent experiments were conducted. All data are shown as the mean ± SD. A comparison between two groups was performed using a student's t-test. ^*^^*^P < .01, ^*^^*^^*^P < .001. ns, not significant. P-value <.05 was considered statistically significant.

As one of the most malignant types of digestive tract cancer cells, PDAC cells have powerful migration and invasiveness capabilities [3]. In this study, we investigated the effect of eravacycline on cell migration abilities. The wound healing assay shows that BxPC-3 cells treated with 10 μM of eravacycline for 0, 24, 48 and 72 h displayed a significantly lower migration rate to a wound introduced in a confluent monolayer of cells than that of the control group (Figure 3). These results indicate that eravacycline inhibited cell migration in human PDAC cells.

### Eravacycline’s effect on the induction of apoptosis in pancreatic cancer cells

**Figure 4:**
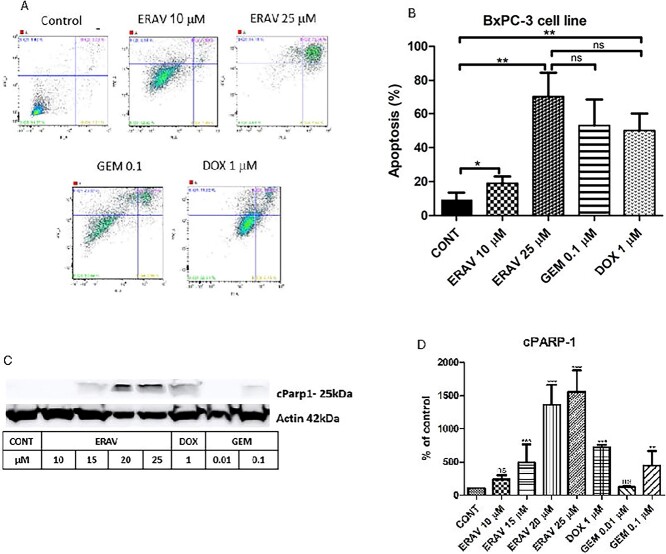
**Eravacycline induces apoptosis in human pancreatic cancer cells**. (A-B) Image and quantification of apoptosis rates of BxPC-3 cells after exposed to control, 1 μM doxorubicin, 0.1 μM gemcitabine, 10 µM eravacycline or 25 µM eravacycline for 72 h by flow cytometry. Statistical analysis of apoptosis intensity in treated cells was performed in vitro. (C-D) BxPC-3 cells after treating with 1 μM doxorubicin, 0.01 μM gemcitabine, 0.1 μM gemcitabine or increasing concentrations of eravacycline for 72 h. The expression of cell apoptosis-related proteins, C-PARP1, was detected using Western blot assay; actin was used as control. At least three independent experiments were conducted. All data are shown as the mean ± SD. A comparison between multiple groups was performed using a one-way ANOVA. ^*^P < .05; ^*^^*^P < .01, ^*^^*^^*^P < .001. ns, not significant. P-value <.05 was considered statistically significant.

To examine eravacycline’s effect on cell growth inhibition and the mechanism underlying this, we investigated the induction of apoptosis activated by eravacycline treatment in BxPC-3 cells by performing an Annexin V-FITC (V-fluorescein isothiocyanate) apoptosis assay. Figure 4 presents the results of our experiment on the effects of the treatment of BxPC-3 cells with increasing concentrations of eravacycline for 72 h, with a positive control of 1 μM of doxorubicin and 0.1 µM of gemcitabine for the same amount of time. This experiment shows that the BxPC-3 cells’ treatment with 10 μM eravacycline resulted in a 3-fold increase in the number of apoptotic cells (Figure 4A and B). Western blot was used to evaluate the C-PARP1, and the results show that C-PARP1 increased significantly after treatment with 15 μM eravacycline compared to the control group (Figure 4C and D). These data indicate that eravacycline influenced apoptosis in BxPC-3 cells.

## DISCUSSION

This study used an ML approach to repurpose existing drugs for anti-cancer use. The results were validated *in vitro*. Our novel approach focuses on identifying molecules with anti-cancer properties instead of focusing on specific predefined targets. We tackled the problem by using Chemprop [12] enriched with drug–drug interaction information, which was not used before for anti-cancer drug repurposing. Drug labels were collected from various sources, and missing information was completed using a lookup mechanism based on chemical similarity.

We used data from various sources to extend Chemprop to consider not only the molecular structure but also to learn from DTI and DDI information [21]. The use of this information significantly improved the model’s accuracy from an AUC of 0.806 to 0.909. We used this model to predict the anti-cancer activity of drugs that have not been tested in cancer research; specifically, we ranked the anti-cancer activity of all FDA-approved molecules in order to detect approved molecules with unknown anti-cancer potential. We analyzed the ranked list to identify the level of correlation between our suggested anti-cancer model’s predictions and the laboratory results obtained in our experiments performed to examine anti-cancer activity on different cancer cells.

In contrast to studies that trace a potential drug by examining small-scale databases containing only a few dozen drugs, our approach was trained on over 1200 molecules; the candidate molecule was found by ranking the full list of approved molecules. A labeled dataset was curated from DrugBank, cancer.com, ClinicalTrials.gov and MeSH. Our initial assessment showed that using a lower dimensionality representation of DTIs and DDIs was beneficial in terms of accuracy. Data from the predictions of the NN were used to identify molecules with potential anti-cancer activity, and the input of the network consisted of a molecule structure. The additional features consistently led to the highest model accuracy. These findings are consistent with previous results from both our lab and other laboratories, which found that target data reflect the mechanisms underlying the structure of drugs with potential anti-cancer activity [28]. To the best of our knowledge, this is the first research to demonstrate the benefit of using DDI information for drug repurposing. We suggest two explanations for the fact that DDI had a better effect on the model’s accuracy than DTI: (1) DDIs of approved drugs are discovered rapidly because patients worldwide take multiple drugs leading to DDI discovery. DTIs, on the other hand, require trials to discover. Hence, DDI information is of higher quality because a missing DDI is more likely to indicate no interaction compared to DTI. In other words, a missing DTI is more likely to represent an undiscovered, existing interaction compared to DDI, and (2) the underlying reason for DDI, for example, the drugs’ pharmacokinetic or pharmacodynamic parameters are highly correlated to anti-cancer activity, and the DDI information indirectly represents them. To limit the effects of missing DDI and DTI values on the model’s performance, we fill in missing values by identifying a similar non-missing molecule based on molecular similarity. For the prediction of molecules’ anti-cancer activity, we report an AUC of 0.909 and AUPR of 0.849. As mentioned in the Results section, our analysis of the models’ predictions for the set of FDA-approved drugs showed that eravacycline was highly ranked, indicative of its potential as a drug capable of inhibiting cancer cell growth. Another reason for selecting eravacycline is that it had not yet been tested for cancer use.

To validate the anti-cancer ML model’s accuracy and discern correlations between predicted drug rankings and actual anti-cancer activity, we conducted *in vitro* experiments. Evaluating eravacycline’s impact on MCF-7, A549, HT-29, BxPC-3 and AsPC-1 cells through XTT assays, we compared it with tigecycline and omadacycline (Figure 2). Omadacycline inhibited cell growth up to 40% after 72 h, at both low and high doses. Tigecycline moderately suppressed proliferation (60–65%) in PDAC cells (BxPC-3, AsPC-1) and lung cancer cells (A549) concentration-dependently within 72 h, while showing no significant effect on MCF-7 and HT-29 cells (Figure 2B). Earlier research on tigecycline at 40 μM demonstrated similar outcomes, achieving around 65% inhibition in BxPC-3 and AsPC-1 cells [9]. In our study, the treatment of BxPC-3 with 25 μM of eravacycline for 72 h suppressed the proliferation in a concentration-dependent manner by up to 90–93% (Figure 2C). These experiments showed conclusively that eravacycline is a potent inhibitor of clonal growth of PDAC cells.

A comparison of the ability of eravacycline and the currently used chemotherapeutic drug, doxorubicin [29, 30], to inhibit pancreatic cancer cell growth was also performed. Doxorubicin is a member of the anthracycline antitumor antibiotic family, where the backbone structure correlates to that of tetracyclines [31, 32]. In the current study, the inhibition rates for the BxPC-3 cell line treated with increasing concentrations of doxorubicin and eravacycline for 72 h were measured by performing XTT assays and found to be 90% for concentrations of 10 and 25 μM, respectively.

To examine eravacycline’s potential to perform its activity through an apoptotic pathway, we examined several signals. The exposure of BxPC-3 cells to eravacycline increased the levels of cleaved PARP1 (C-PARP1), indicating the activation of apoptotic cell death. Additionally, to investigate whether the proliferation of BxPC-3 cells inhibited by eravacycline is related to the progression of the cell cycle, we examined the cell cycle progression by performing flow cytometry. The apoptosis induction activated by eravacycline treatment in BxPC-3 pancreatic cancer cells using Annexin V-FITC resulted in significantly increased apoptosis. The mechanisms by which eravacycline mediates its antiproliferative effects have not been fully elucidated.

## CONCLUSIONS

Our study showed that the use of a deep learning model based on a state-of-the-art NN enriched with DDI information enabled the identification of eravacycline, which was shown to have very potent anti-cancer activity in *in vitro* experiments. Since growth inhibition and apoptosis are important targets in cancer research, the growth inhibitory and proapoptotic effects of eravacycline, *in vitro*, may be exploited in future research for the development of an effective clinical anti-cancer therapy. Our research shows that ML drug repurposing model can be applied on a wide range of new oncology therapeutics, for potential direct clinical translation.

Code: https://github.com/eyalmazuz/DrugRepurposing

**Key Points**

- Eravacycline, an antibacterial drug, shows promising anti-cancer effects in treating pancreatic ductal adenocarcinoma.
- Machine learning models, particularly deep neural networks using drug–target and drug–drug interaction data, were used to repurpose eravacycline.
- *In vitro* study reveals that eravacycline inhibits the proliferation and migration of BxPC-3 pancreatic cancer cells and induces apoptosis.
- The study demonstrates the potential of using machine learning for drug repurposing in cancer therapy.

## Supplementary Material

Supplementary_Information_ERAVACYCLINE_bbae108

## Author Biographies

**Adi Jabarin** is a student in the Department of Biochemistry and Pharmacology at Ben-Gurion University of the Negev (BGU). His primary research interests lie in the field of drug development, with a particular focus on medicinal chemistry, pharmacological research, and the comprehensive drug development process. Adi's expertise extends from the initial stages of drug design and synthesis to the crucial phases of optimization, target identification, and mechanistic studies.

**Guy Shtar** received the MSc and PhD degrees in information systems and software engineering from BGU. His main research topics are in drug knowledge discovery using machine learning (ML) and adversarial ML.

**Valeria Feinshtein**, lab manager, received the PhD degree in pharmacology, in human placental barrier research—drug resistance proteins and cannabidiol (CBD). Work in the field of medicinal chemistry, pharmacological research of human eye outflow system, exosomes, pharmacist, drug delivery research and cannabinoids.

**Eyal Mazuz** received the PhD degree in ML and artificial intelligence, research application of transformer models and topics drug discovery and molecule generation.

**Bracha Shapira** is the professor at BGU’s Department of Software and Information Systems Engineering. She has been both the department chair and vice dean for research. She leads the Recommender Systems Lab and is a research scientist at the eBay R&D center in Israel**.**

**Shimon Ben-Shabat** is the professor and leads research in bioorganic & medicinal chemistry and pharmacology, focusing on innovative drug development projects. His work in bioorganic and medicinal chemistry involves design, synthesis, drug delivery, targeting, mechanistic studies and bio-analytical research.

**Lior Rokach** is the full professor of information systems and software engineering. He was the chair of the Department of Software and Information System Engineering at BGU. Rokach’s research focuses on data science, ML, big data, deep learning and data mining.
