# Supplementary material for: Eravacycline, an antibacterial drug, repurposed for pancreatic cancer therapy: insights from a molecular-based deep learning model
Source: Brief Bioinform. 2024 Apr 21;25(3):bbae108. doi: 10.1093/bib/bbae108 (PMC11033730; doi:10.1093/bib/bbae108)
Supplement: Supplementary_Information_ERAVACYCLINE_bbae108 [file supplementary_information_eravacycline_bbae108.docx]

**Supplementary Information**

**Eravacycline, an Antibacterial Drug, Repurposed for Pancreatic Cancer Therapy: Insights from a Molecular-Based Deep Learning Model**

Adi Jabarin^1^**, Guy Shtar^2^**, Valeria Feinshtein^1^, Eyal Mazuz^2^, Bracha Shapira^2^, Shimon Ben-Shabat^1*^, Lior Rokach^2*^.

^1^Department of Clinical Biochemistry and Pharmacology, Ben-Gurion University of the Negev, P.O.B. 653, Beer-Sheva 8410501, Israel.

^2^Department of Information Systems and Software Engineering, Ben-Gurion University of the Negev, P.O.B. 653, Beer-Sheva 8410501, Israel.

* Corresponding authors at: Ben-Gurion University of the Negev, Beer-Sheva, Israel.

*E-mail address*: [sbs@bgu.ac.il](mailto:sbs@bgu.ac.il) (S. Ben-Shabat) and [liorrk@post.bgu.ac.il](mailto:liorrk@post.bgu.ac.il) (L. Rokach)

** Equal contribution & first authorship:

These authors have contributed equally to this work and share the first authorship -

Adi Jabarin and Guy Shtar

**Table of contents**

**Supplementary Methods** 2-5

S1. Training set creation 2

S2. Handling imbalanced data 2

S3. Feature engineering 2-3

S4. Message passing neural network 3

S5. Cell culturing 3

S6. Tetracycline derivative treatment 3-4

S7. Cell proliferation assay 4

S8. Determination of half maximal inhibitory concentration (IC50) of

eravacycline, tigecycline, and omadacycline 4

S9. Cell migration and scratch wound healing assay 4

S10. Flow cytometry analysis 4

S11. Protein extraction from Bxpc-3 cells 5

S12. Western blot analysis 5

**Supplementary results** 5-8

Supplementary Figures 5-6

Table S1 6

S13. Pharmacologist-Driven Drug Candidate Selection 6-8

**References** 8

**Supplementary Methods**

**S1.** **Training set creation**

The training set was curated from DrugBank. We used only molecules by removing biologic drugs, the Simplified Molecular Input Line Entry System (SMILES) was extracted from the DrugBank record. Positive samples were identified from ClinicalTrials.gov and cancer.com, along with the drugs' associated MeSH terms. Our assumption was that a negative drug (not anti-cancer) can be discovered to be a positive one (anti-cancer) in the future, which means the negative samples are weakly labeled compared to the positive ones. Therefore, we curated a negative set which currently has no evidence of being anti-cancer but has enough history about it, implying that the scientific community had opportunities to discover any anticancer activity. We labeled 451 positive drugs and 918 negative drugs; however, some SMILES representations were not parsed correctly by Chemprop and were removed. The final train set contained 408 positive drugs and 903 negative drugs. Our GitHub repository contains the labeled dataset, including the filtering information described in this subsection.

**S2. Handling** **imbalanced data**

Our training data is somewhat imbalanced, having about double the number of negative samples compared to positive. Class imbalance can be addressed with three main strategies: 1) data pre-processing, such as oversampling, undersampling, and generating synthetic data; 2) algorithmic adjustments, such as class weighting in the loss function; and 3) hybrid approaches, which combine various approaches.^1^ As part of our experiments, we focused on the first and last approaches. For pre-processing, we tried undersampling the negative class, specifically, we sampled 800 of the 918 negative drugs. As a hybrid approach, we used the *class_balance* argument in Chemprop which samples the same amount of the minority class in each batch. The results showed little to no change and therefore we did not continue our research in this direction.

**S3.** **Feature engineering**

The DTI and DDI data were collected from DrugBank 5.1.8. We converted these data into a binary representation, the two tables represented the existence or absence of an interaction. The interaction data is sparse due to the large number of possible interactions and partial information currently known; some currently unknown interactions will be discovered in the future. To deal with the sparsity, we reduced the dimensionality of both matrices. The DTI data were processed using PCA with a predefined representation (embedding) size of 64, this parameter was not tuned. The 64 dimensions capture 45% of the variance of the original matrix. The DDI can also be compressed using PCA, but to exploit the fact that the DDI matrix is symmetric, we used AMFP, a method we previously developed. AMFP compresses the adjacency matrix using a shallow neural network. The neural network uses a shared representation for both drugs. In other words, the rows and columns share the same embedding. Following this step, AMFP propagates the representation of interacting drugs. The second step results in averaging each drug's original embedding with the embedding of the drug's interacting drugs.

DrugBank does not contain interaction information for all drugs. We imputed these data using nearest neighbor search based on molecular similarity. The DrugBank website allows users to search for drugs with similar structures; this feature was used to identify similar drugs. The minimum default threshold in the search is 0.7, if the search did not return any result, we used the average embedding representation. Three hundred forty-four drugs used the average embedding for DTI. One hundred eighty-nine drugs used the average embedding for DDI.

**S4. Message passing neural network**

We base our work on Chemprop, which utilizes a hybrid representation in deep neural networks for accurate molecular property prediction. Chemprop employs a Directed Message Passing Neural Network (D-MPNN) approach, focusing on bond-centric convolutions. This method efficiently represents molecular structures and addresses the limitations of data scarcity and local feature representation. In the readout phase of Chemprop, the model synthesizes the accumulated information from the molecular graph. To enhance the model's capability, we integrate features encompassing drug-target interactions and drug-drug interactions, represented as vectors for each molecule. The interactions are incorporated in the following way:

$$\hat{y}=f(cat\left( h,h_{dti},h_{ddi} \right))$$

Where $\hat{y}$ is the predicted binary outcome, $f$ is a feed forward neural network, $h$ is the molecule feature vector which is learned by the D-MPNN model, $h_{dti}$ is the DTI embedding learned by applying PCA to the DTI data, and $h_{ddi}$ is the DDI information learned by AMFP. The network is trained end-to-end with binary cross-entropy loss.

For balanced scaffold-based split, we used Chemprop's implementation. Same splits were used for all models including baselines.

**S5. Cell culturing**

All cell lines were obtained from ATCC. Human PDAC (AsPC-1, BxPC-3), breast (MCF-7), lung (A549), and colon (HT-29) cancer cell lines, obtained from ATCC, were cultured in DMEM (MCF-7, A549, HT-29) or RPMI-1640 (BxPC-3, AsPC-1) media. Media contained 10% FBS, 200 μM L glutamine, and 1% penicillin-streptomycin, and cells were maintained at 37°C in a 5% CO2 humidified incubator.

**S6.** **Tetracycline derivative treatment**

Eravacycline dihydrochloride (MedChem Express (MCE)) was dissolved in Deuterium depleted water (DDW). tigecycline (MCE) and omadacycline (MCE) were dissolved in dimethyl sulphoxide (DMSO). All drugs were prepared as 100 mM stock solutions. All cell lines were treated with DMSO as a control (less than 0.1%) or tigecycline, omadacycline, or eravacycline (at the indicated concentrations) for 72 hours. Cell viability and proliferation were determined by using the XTT Cell Proliferation Assay Kit (Promega).

**S7. Cell proliferation assay**

Inhibition of cell proliferation was determined by using the XTT Cell Proliferation Assay Kit. Cells (4,000 cells/well) attached overnight in 96-well plates in a 5% CO2, 37°C incubator. Following attachment, cells were exposed to varying concentrations (1-50 μM) of tigecycline, omadacycline, or eravacycline in culture medium for specified durations. Subsequently, 50 μl of XTT solution was added per well and incubated at 37°C for two hours. Absorbance was measured at 450 nm (Bio-Rad iMark) and reference wavelength of 655 nm.

**S8. Determination of half maximal inhibitory concentration (IC50) of**

**eravacycline, tigecycline, and omadacycline**

To evaluate the half maximal inhibitory concentration (IC50) of tetracycline derivatives on five different cell lines (MCF-7, A549, HT-29, BxPC-3, and AsPC-1) were seeded and allowed to attach overnight. All cells were washed with phosphate buffered saline (PBS) and then cultured in fresh media containing the drugs tigecycline, omadacycline, or eravacycline at an increasing concentration (0 -50 μM) for 72 h. Inhibition of cell proliferation was determined by using the XTT Cell Proliferation Assay Kit.

**S9. Cell migration and scratch wound healing assay**

BxPC-3 cells reached confluence in six-well plates, followed by creating a scratch using a 200μl pipette tip. After washing with PBS to remove debris, cells were exposed to medium alone or with 10 μM eravacycline. Cell migration was monitored using a Nikon ECLIPSE Ts2 fluorescence microscope, capturing images, and measuring wound closure rates at specified intervals.

**S10. Flow cytometry analysis**

Cells were cultured in 6-well plates with varying eravacycline, gemcitabine, or doxorubicin concentrations for 72 h; medium served as the control. Post-incubation, trypsinized cells underwent flow cytometry analysis. After resuspension in 100 μl binding buffer, cells were stained with 5 μl PI and 5 μl Annexin V-FITC for 20 min in the dark. Analysis was performed using the Sony SP6800 Spectral cell analyzer and accompanying software.

**S11. Protein extraction from BxPC-3 cells**

Following 72 h incubation of BxPC-3 cells with medium alone as a control or medium containing different concentrations of eravacycline, gemcitabine, or doxorubicin, BxPC-3 cells were harvested and lysed, as previously described.^2^ briefly, BxPC-3 cells were lysed in a buffer consisting of HEPES, NaCl, EGTA, EDTA, glycerol, MgCl2, Triton X-100, and protease phosphatase inhibitors. After incubation and sonication, lysates underwent centrifugation, yielding supernatants for protein concentration analysis via the Bradford assay at 595 nm using a Bio-Rad iMark Microplate Absorbance reader.

**S12. Western blot analysis**

For cell apoptosis-related protein expression analysis, Western blotting targeted C-PARP1 using Actin as a control. Samples (20 μg protein) mixed with Laemmli buffer were separated on 10% SDS-PAGE, transferred to a nitrocellulose membrane, and probed with primary anti C-PARP1 antibodies overnight. Chemiluminescence detection with ImageQuant LAS 500 and semi-quantitative analysis via Mac Biphotonic ImageJ software were employed.

**Supplementary Results**

**
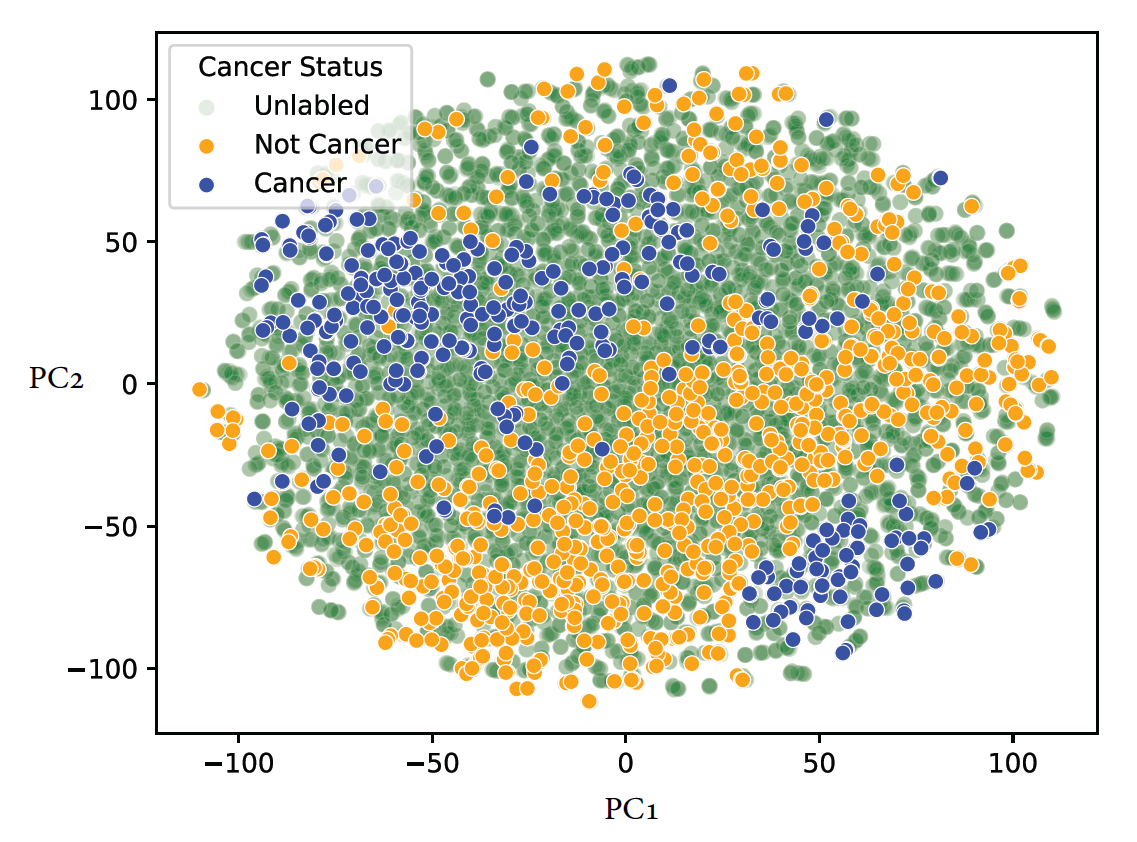
**

**Figure S1.** A t-distributed Stochastic Neighbor Embedding (t-SNE) analysis of our data. The original high dimensional data is the molecule feature vector which is learned by the D-MPNN model.


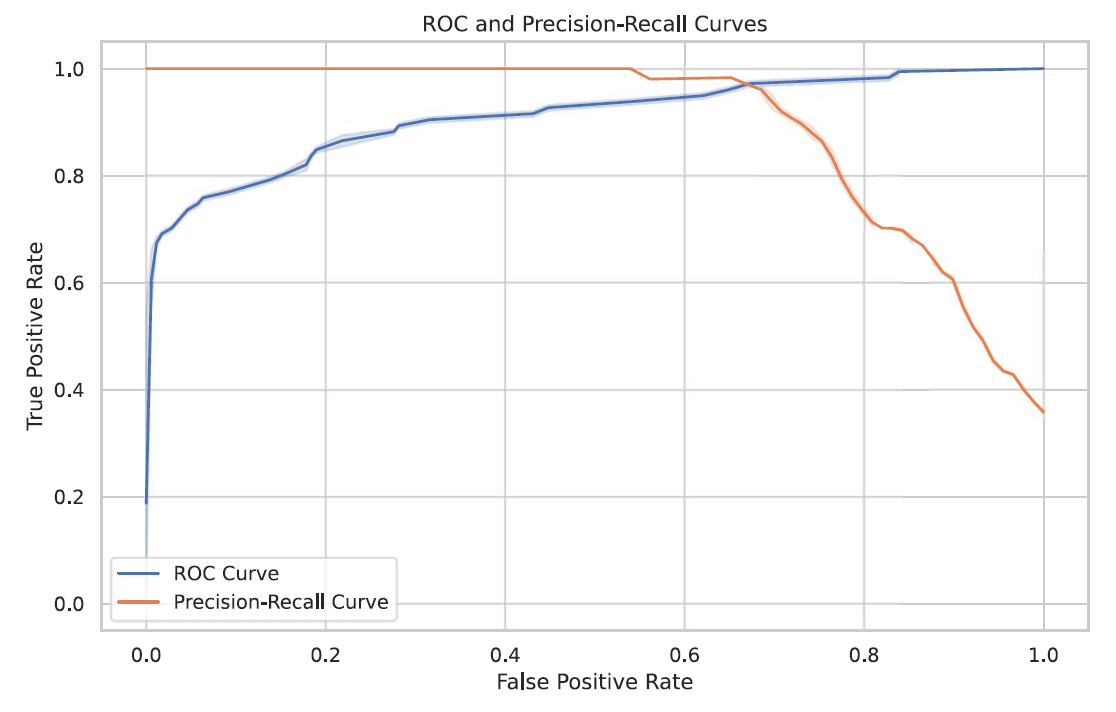


**Figure S2.** Receiver Operating Characteristic (ROC) and Precision-Recall curves of Chemprop model trained with DDI and DTI data.

|  | | | **Predicted** | | |
| --- | --- | --- | --- | --- | --- |
|  |  |  | **True** | **False** |  |
| **True** | **True** | 163 | | 11 | |
|  | **False** | 21 | | 68 | |

**Table S1.** Confusion matrix for the test set evaluated with a Chemprop model which was trained with DDI and DTI data.

**S13. Pharmacologist-Driven Drug Candidate Selection**

We would like to emphasize that the pharmacologist has been actively involved in all stages of the machine learning (ML) process, including data curation, model development, and performance evaluation. The decision-making process involves a careful evaluation of various factors, including but not limited to:

- **Biological Relevance:**

The pharmacologist assessed the biological context to ensure predicted candidates aligned with known anti-cancer pathways and mechanisms. For instance, eravacycline has family members such as tigecycline, ninocycline, and doxycycline, which have demonstrated anti-cancer activity in previous studies.^2,3^ This consideration of drug family history enhanced the selection process, leveraging insights from the documented anti-cancer properties of these family members. A detailed comparison of structural and biochemical properties with effective family members further refined the selection criteria.

- **Tetracyclines and Anti-Cancer Activity:**

Consider tetracyclines, discovered in the late 1940s; they are broad-spectrum antibiotics used to this day for treating infectious diseases. Tetracyclines exert their antimicrobial effect by inhibiting protein translation through binding to the bacterial 30S ribosomal subunit, blocking aminoacyl-tRNA attachment to the A-site and preventing protein synthesis. Previous studies indicate that tetracyclic analogs induce apoptosis in various cancer cell lines (prostate, breast, gastric cancer, leukemia, melanoma, osteosarcoma, colorectal, and pancreatic cancer). Some studies highlight the therapeutic potential of doxycycline, minocycline, and chemically modified tetracycline-3 (COL-3) in cancer treatment. Recent investigations suggest tigecycline, a new tetracycline member, may serve as an adjunct to cancer treatment, particularly against drug-resistant pathogens.

- **Tetracyclines' Chemical Structure and Modifications:**

The chemical structure of tetracyclines comprises a linear fused tetracyclic ring (A-D) with various attached pharmacophores. Structural modifications at the C7 and C9 positions, particularly tail extensions, have led to the discovery of tigecycline, omadacycline, and eravacycline. These drugs share a unique tail extension at the C9 position. The similarity in the tail structure between eravacycline and tigecycline is notable, with both molecules featuring an acidic hydrogen on the amide nitrogen and a lipophilic tail extension. This commonality contributes to greater lipid solubility in both compounds. Notably, eravacycline differs from tigecycline and omadacycline due to a fluorine atom at the C7 position. This structural distinction, alongside the absence of a hydroxyl group at the C6 position, enhances lipid solubility, possibly explaining eravacycline's superior anti-cancer activity compared to tigecycline and omadacycline.

- **Fluorine Atom in eravacycline and Anti-Cancer Activity:**

The essential difference between eravacycline and tigecycline/omadacycline is the fluorine atom at the C7 position. This modification in eravacycline, as suggested in prior studies on tetracyclic analogs, may potentiate its anti-cancer activity. Furthermore, strategic integration of fluorine in drug development has been well-explored for enhancing potency and metabolic stability in anti-cancer substances. Fluorinated anti-cancer agents like fludarabine, clofarabine, and tezacitabine have shown efficacy in cancer treatment. Therefore, the fluorine atom at the C7 position of eravacycline may partially explain its superior anti-cancer activity compared to tigecycline or omadacycline.

- **Experimental Feasibility:** Practical considerations, including compound availability and alignment with experimental protocols, were carefully assessed to facilitate the transition from in-silico predictions to experimental validation. Essential pharmacokinetic properties and factors like drug solubility in water, as detailed in pertinent literature, significantly influenced the practical feasibility of experimentally validating repurposed drugs.
- **Risk Assessment:** The pharmacologist evaluated potential risks associated with each candidate, considering factors such as toxicity profiles, off-target effects, and other safety considerations. This risk assessment was informed by an in-depth examination of relevant literature and an analysis of the preclinical and clinical phases of research pertaining to the candidate drugs. Specifically, eravacycline, despite being subject to clinical trials, did not show a high incidence of serious adverse events or discontinuation, contributing positively to its risk profile.^4^

**Supplementary References**

1 Rezvani, S. & Wang, X. A broad review on class imbalance learning techniques. *Applied Soft Computing* **143**, 110415, doi:10.1016/j.asoc.2023.110415 (2023).

2 Huang, S. W. *et al.* Cancer as an infectious disease: A different treatment alternative using a combination of tigecycline and pyrvinium pamoate - An example of breast cancer. *J Microbiol Immunol Infect* **55**, 51-59, doi:10.1016/j.jmii.2020.12.008 (2022).

3 Song, H., Fares, M., Maguire, K. R., Siden, A. & Potacova, Z. Cytotoxic effects of tetracycline analogues (doxycycline, minocycline and COL-3) in acute myeloid leukemia HL-60 cells. *PLoS One* **9**, e114457, doi:10.1371/journal.pone.0114457 (2014).

4 Meng, R. *et al.* The efficacy and safety of eravacycline compared with current clinically common antibiotics in the treatment of adults with complicated intra-abdominal infections: A Bayesian network meta-analysis. *Front Med (Lausanne)* **9**, 935343, doi:10.3389/fmed.2022.935343 (2022).
